# Ubiquitous Aberration in Cholesterol Metabolism across Pancreatic Ductal Adenocarcinoma

**DOI:** 10.3390/metabo12010047

**Published:** 2022-01-07

**Authors:** Venugopal Gunda, Thiago C. Genaro-Mattos, Jyoti B. Kaushal, Ramakanth Chirravuri-Venkata, Gopalakrishnan Natarajan, Kavita Mallya, Paul M. Grandgenett, Karoly Mirnics, Surinder K. Batra, Zeljka Korade, Satyanarayana Rachagani

**Affiliations:** 1 Department of Biochemistry and Molecular Biology, University of Nebraska Medical Center, Omaha, NE 68198, USA; venu.gunda@unmc.edu (V.G.); jyoti.kaushal@unmc.edu (J.B.K.); r.chirravurivenkata@unmc.edu (R.C.-V.); g.natarajan@unmc.edu (G.N.); kmallya@unmc.edu (K.M.); sbatra@unmc.edu (S.K.B.); 2 Munroe-Meyer Institute for Genetics and Rehabilitation, University of Nebraska Medical Center, Omaha, NE 68106, USA; thiago.mattos@unmc.edu (T.C.G.-M.); karoly.mirnics@unmc.edu (K.M.); 3 Eppley Institute for Research in Cancer and Allied Diseases, University of Nebraska Medical Center, Omaha, NE 68198, USA; pgrandgenett@unmc.edu; 4 Department of Pathology and Microbiology, University of Nebraska Medical Center, Omaha, NE 68198, USA; 5 Fred and Pamela Buffett Cancer Center, University of Nebraska Medical Center, Omaha, NE 68198, USA; 6 Department of Pediatrics, Biochemistry and Molecular Biology, College of Medicine, University of Nebraska Medical Center, Omaha, NE 68198, USA; zeljka.korade@unmc.edu

**Keywords:** PDAC (pancreatic ductal adenocarcinoma), free cholesterol, Dhcr24, Dhcr7, sterol analysis

## Abstract

Pancreatic cancer (PC) is characterized by metabolic deregulations that often manifest as deviations in metabolite levels and aberrations in their corresponding metabolic genes across the clinical specimens and preclinical PC models. Cholesterol is one of the critical metabolites supporting PC, synthesized or acquired by PC cells. Nevertheless, the significance of the de novo cholesterol synthesis pathway has been controversial in PC, indicating the need to reassess this pathway in PC. We utilized preclinical models and clinical specimens of PC patients and cell lines and utilized mass spectrometry-based sterol analysis. Further, we also performed in silico analysis to corroborate the significance of de novo cholesterol synthesis pathway in PC. Our results demonstrated alteration in free sterol levels, including free cholesterol, across in vitro, in vivo, and clinical specimens of PC. Especially, our sterol analyses established consistent alterations in free cholesterol across the different PC models. Overall, this study demonstrates the significance and consistency in deviation of cholesterol synthesis pathway in PC while showing the aberrations in sterol metabolite intermediates and the related genes using preclinical models, in silico platforms, and the clinical specimens.

## 1. Introduction

Cholesterol is a vital structural component of cellular membranes and plays a key role in various biological processes involving physiology and disease progression, including cancer [1,2]. Due to the cellular utilization of free cholesterol as a membrane constituent and an intermediate in sterol metabolism, free cholesterol pools become significantly decreased in cells, thus leading to cellular dependence on cholesterol replenishment for vital processes [3]. Such striking reliance on cholesterol replenishment, including the de novo biosynthesis of cholesterol, is noticed in malignancies, wherein cancer cells exhibit prolific division and migration potential, and abnormally rely on cholesterol to maintain their proliferative and migratory competence [2,4,5,6]. Such aberrant cholesterol dependence often manifests as deviations in abundance of cholesterol and the intermediates of the cholesterol synthesis pathway in cancer tissues [3,7].

Pancreatic cancer (PC) is characterized by rapid growth evident at dismal stages of the disease, and pancreatic tumor cells exhibit consistent proliferative and migration potential at early as well as at dismal stages of cancer [8,9]. Oncogenic mutations, including the most frequent *KRAS*^G12D^ mutation, promote the proliferative and migratory capacity in PC cells [10]. In addition to the stimulus provided by the oncogenes, PC cells also require oncogene-driven metabolism, of which cholesterol metabolism plays a crucial role in the pathological phenotype of PC [11,12]. Cholesterol metabolism impacts survival in both the clinical and preclinical contexts of PC [12]. Independent studies supported the notion of PC’s dependence on cholesterol through in vitro, in vivo, and in silico analyses [11,12]. While uptake of cholesterol for in vitro growth by PC cells was established [13]; deletion of cholesterol metabolic genes also showed cytostatic effects on *Kras*-driven tumor growth, in vivo in mouse PC models [12]. Furthermore, retrospective studies revealed poor survival outcomes in PC patients associated with sterol metabolic genes [12,14]. Thus, in vitro and in vivo models of PC reveal an inevitable association between cholesterol synthesis and growth, survival, as well as therapy response of PC, which is strongly supported by retrospective survival analyses [12].

We have previously shown that cholesterol metabolism plays a vital role in the radiation resistance of PC, and blockage of cholesterol synthesis sensitizes PC to radiation [15]. Based on the previous observations indicating dependence of PC on cholesterol synthesis pathway, we hypothesized that free cholesterol and cholesterol synthesis intermediates alter across different models of PC. Quantification of free cholesterol and sterol intermediates in cholesterol biosynthesis become critical to unveil the pathological role of cholesterol metabolism in disease [16,17], which is pertinent to our hypothesis. Such quantitation of cholesterol and its upstream metabolites preferably enumerates the fluctuations in cholesterol synthesis associated with disease, including cancers [16,18]. However, discrepancies in sterol content across biological models [19] and the lack of comparative analyses of cholesterol metabolites from a range of malignant specimens, including the in vitro, in vivo, and clinical specimens [20] of a single cancer model, impede the possibility of correlating the metabolite readouts across multiple specimen platforms within cancer.

In the current study, we have utilized the in silico analysis, clinical specimens, and in vitro and in vivo models of pancreatic ductal adenocarcinoma for enumerating the aberrant cholesterol synthesis pathway in PC. Our study sheds light on the consistency in the deviation of cholesterol abundance and its synthesis across the biological specimens of PC. We focused on the alterations in gene expression patterns involved in cholesterol synthesis pathway, quantification of free sterols comprising cholesterol synthesis pathway, and the prognostic outcomes of the genes involved in cholesterol synthesis pathway in PC.

## 2. Results

### 2.1. Higher Expression of Cholesterol Synthesis Genes in Human Pancreatic Ductal Adenocarcinoma

**Figure 1:**
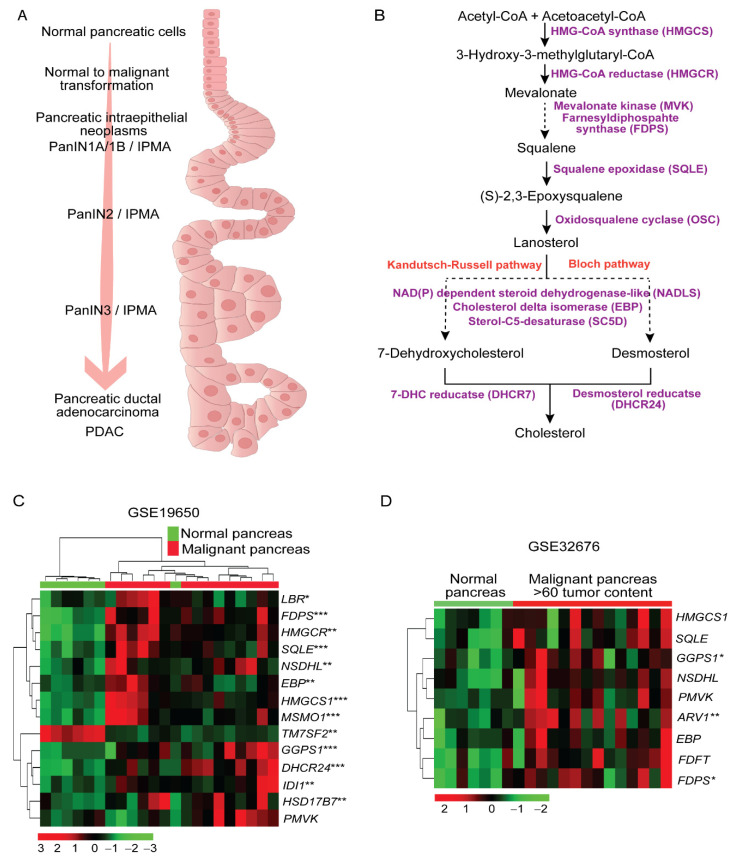
Changes in cholesterol synthesis pathways in pancreatic cancer. (**A**) A schematic of pancreatic cancer progression. (**B**) List of some of the enzymes and metabolic intermediates involved in the cholesterol biosynthesis pathway. (**C**,**D**) Heatmaps of alterations in cholesterol synthesis enzymes in pancreatic cancer. Color gradient: red, black, to green in each row of the heatmaps indicates relative differences in the gene expression patterns as indicated by the scale provided below the heatmap. The significant changes are marked with * <0.05; ** <0.01; *** <0.001.

Pancreatic malignancies are histologically well-characterized exocrine lesions that can progress from three precursor lesions, namely, intraductal papillary mucinous-neoplasms (IPMNs), intraductal papillary-mucinous adenomas (IPMAs), and pancreatic intraepithelial neoplasms (PanINs), to pancreatic ductal adenocarcinoma (PDAC) through oncogenic transformation and mutations in tumor suppressor genes (Figure 1A). Along with histological abnormalities, malignant transformation also drives metabolic deviations in PC, as evident in the mouse models of PC [21,22]. Anabolism of cholesterol constitutes a series of regulated biosynthesis steps, which have been least studied in the progression of PC. Cholesterol biosynthesis initiates with Acetyl-CoA followed by sequential intermediates comprising hydroxymethylglutaryl-CoA (HMG-CoA), mevalonate, farnesylpyrophosphate, lanosterol, desmosterol, and 7-dehydrocholesterol (7-DHC), to name a few intermediates (Figure 1B). In our analysis, human pancreatic tumors and premalignant lesions exhibited alterations in gene expression patterns related to the enzymes involved in the cholesterol synthesis pathway as shown in Figure 1C,D. As presented in heatmaps, the expression patterns of sterol synthesis enzymes such as hydroxymethylglutaryl-CoA syntahse-1 (HMGCS1), 3-hydroxy-3-methyl-glutaryl-coenzyme A reductase (HMGCR), squalene epoxidase (SQLE), NAD(P)-dependent steroid dehydrogenase-like (NSDHL) protein, β-hydroxysteroid-d(8),d(7)-isomerase (EBP), and 24-dehydrocholesterol reductase (DHCR24) were significantly upregulated in the human pancreatic tissues of malignant cohorts compared to normal pancreas (Figure 1C,D). Relative expression patterns obtained from the dataset GSE19650 revealed differences in expression of cholesterol synthesis genes among the normal, premalignant (IPMNs and IPMAs), and PDAC categories (Figure 1C). Furthermore, evaluating the difference between normal and malignant pancreatic tissues with higher tumor cellularity revealed consistent upregulation of genes involved in cholesterol synthesis in PC (Figure 1D). Nevertheless, there is significant upregulation of 13 cholesterol biosynthetic pathway genes in the pancreatic cancer compared to cancer-adjacent normal tissues (Figure 1C) in the GSE19650 dataset. When we compared pancreatic cancer tissue with high cellularity, we observed only three genes were significantly overexpressed in a PC patient’s tissues compared to cancer-adjacent normal tissues (GSE32676) (Figure 1D).

### 2.2. Sterol Alterations in Human Pancreatic Tumors

**Figure 2:**
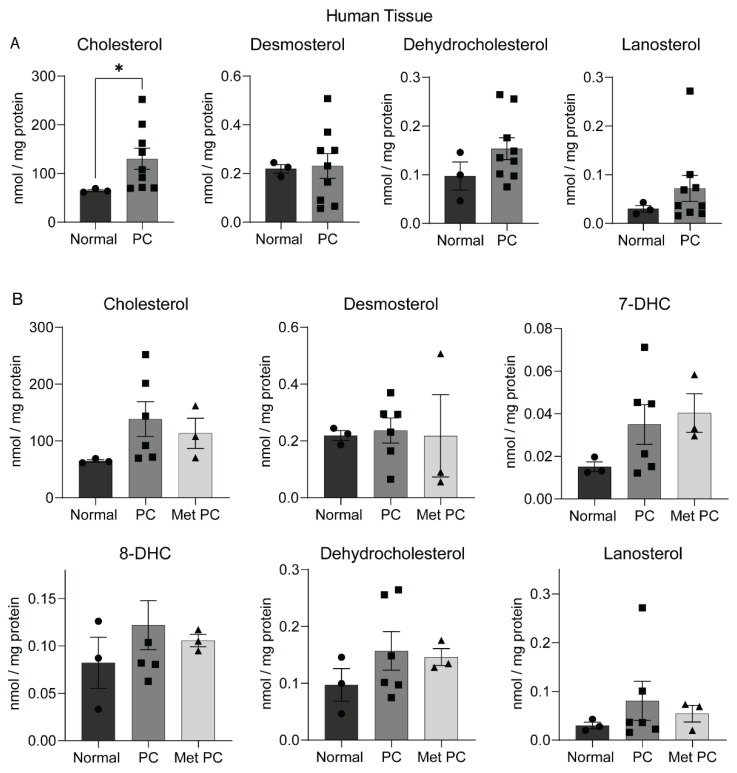
Sterol changes in human pancreatic tissues. (**A**) Mean and standard error plots showing sterol levels in normal human pancreatic tissues and pancreatic cancer tumors (PC) normalized with the protein content of respective tissue specimen. (**B**) Mean and standard error plots showing sterol levels in normal human pancreatic tissues, pancreatic tumors (PC), and metastatic pancreatic cancer (Met PC) tissues normalized to protein content of the respective tissue specimen. * = 0.0622, indicates significant difference in cholesterol comparing normal vs. PC obtained through Welch’s *t*-test.

To determine if the changes in the gene expression (Figure 1C,D) translate into changes in sterols, we measured the levels of sterols in human pancreatic tissues using LC–MS/MS method. Our method included the comparative analysis of unesterified free sterols from the patient tumors as well as from the cancer-adjacent normal pancreas. We found a lack of significant alteration in levels of cholesterol intermediates lanosterol, desmosterol, and dehydrocholesterol between the normal and tumor specimens. However, free cholesterol content was significantly elevated in pancreatic tumors compared to the cancer-adjacent normal pancreas (Figure 2A). Pancreatic cancer patient specimens often contain premalignant zones reflecting distinct stages of tumor development, primary tumors, and the metastatic tissues from distant organs [8]. Therefore, we compared the levels of free sterols among the patient specimens comprising the primary and metastatic sites of collection. Apart from the cholesterol, which was higher in pancreatic tumors compared with normal tissues, other sterols analyzed in this pathway did not exhibit significant differences among the normal, primary malignant pancreatic tissues, and metastatic tissues (Figure 2A,B), this may be due to small sample size used in the current study.

### 2.3. An Elevated Level of Serum Cholesterol in Human Pancreatic Cancer

**Figure 3:**
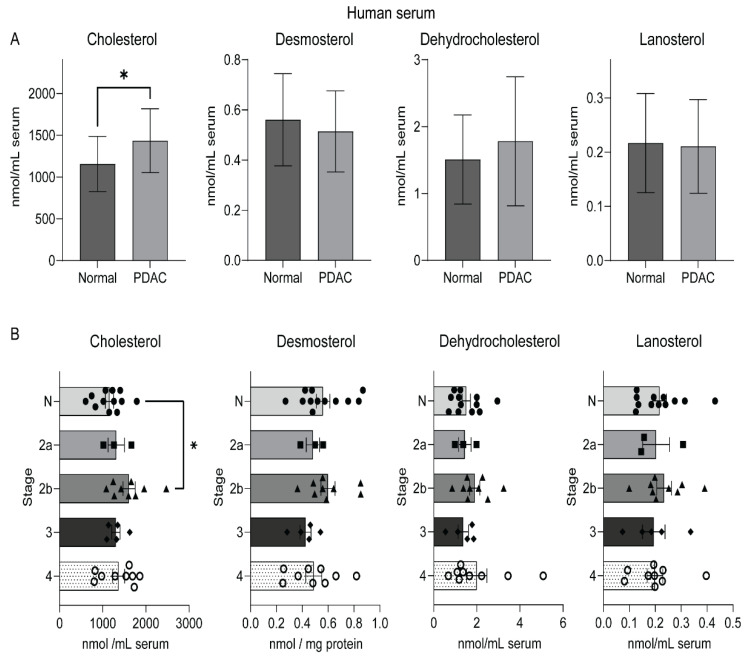
Serum cholesterol levels are high in human pancreatic cancer. (**A**) Mean and standard deviation plots showing sterol levels in human sera collected from healthy (normal) and patients with pancreatic ductal adenocarcinoma (PDAC) normalized with the volume of the specimen. (**B**) Mean and SE plots showing sterol levels in human normalized to serum volume and classified based on healthy (normal) and stagewise progression of pancreatic cancer, including stages 2a, 2b, 3, and 4, respectively. * indicates *p* < 0.05 obtained through unpaired *t-*test.

Serum constitutes the source and sink for multiple metabolites, including cholesterol, enabling noninvasive analysis of cholesterol in pathological scenarios, including cancer [23]. We performed sterol analysis using sera to verify if high free cholesterol levels in human PC tumors are reflected in sera of the PC patients. Indeed, our analyses using sera from PC patients showed significantly higher un-esterified cholesterol levels compared with normal samples (Figure 3A). However, lanosterol, desmosterol, and dehydrocholesterol did not differ significantly among these groups, this may be due to small sample size. We further categorized the serum samples from PC patients into subtypes based on the stage of patients during diagnosis. Our analyses revealed a significant increase in the level of cholesterol in the PC stage 2B patient samples compared to the sera from healthy individuals. Such a significant difference was not noticed in serum cholesterol content from other stages of PC (2a, 3, and 4, respectively) in comparison to the normal serum samples (Figure 3B). This result may be due to the very small cohort used in this study, and precise interpretation of current outcomes in future studies is required to validate these findings.

### 2.4. Sterol Upregulation in Different Mouse Models of PDAC

**Figure 4:**
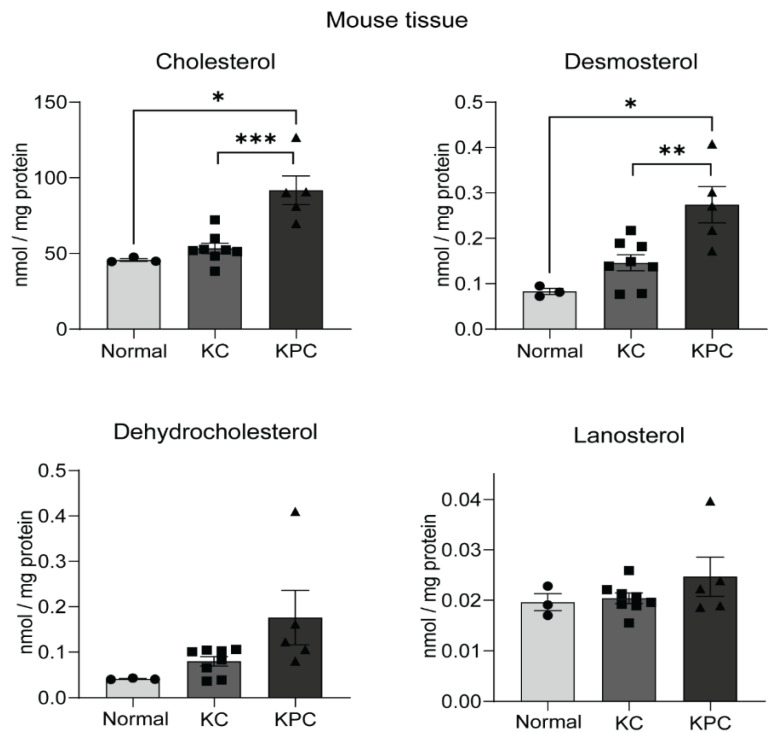
Sterol levels increase in GEMM models of pancreatic cancer: Mean and standard deviation plots showing sterol levels in normal, *Kras*^G12D^;*PdxCre* (KC) and *Kras*^G12D^;*Trp53*^R172H^;*PdxCre* (KPC) pancreatic tissues. *, ** and *** indicate *p* < 0.05, 0.01 and 0.001 respectively, obtained through unpaired *t-*test.

Genetically engineered mouse models (GEMMs) were developed with either the pancreas-specific expression of oncogenic *Kras*^G12D^ through *PdxCre* (KC), or activation of *Kras*^G12D^ in combination with *Trp53*^R172H^ through *PdxCre* (KPC). We observed the development of PanIN lesions at 5 and 10 weeks of age and subsequently cancer with metastases at 25 and 50 weeks of age in KC and KPC models, respectively. These mice models for PC recapitulate the human PC in the defined genetic and histopathological manner [24]. We utilized the pancreas from normal, *Kras*^G12D^;*PdxCre* (KC) and *Kras*^G12D^;*Trp53*^R172H^;*PdxCre* (KPC) mice models and analyzed levels of free cholesterol and other sterols. We observed significantly high cholesterol and desmosterol in mouse pancreatic tumor tissues collected from KPC mice models compared to normal as well as KC mice (Figure 4).

### 2.5. Sterol Levels from In Vitro Models of PC

Human pancreatic cancer cell lines reveal dependence of pancreatic cancer on cholesterol metabolism in a therapeutic context [15]. However, sterol content could be altered through either uptake or intracellular synthesis [25]. Therefore, we evaluated the sterol content in PC cell lines.

**Figure 5:**
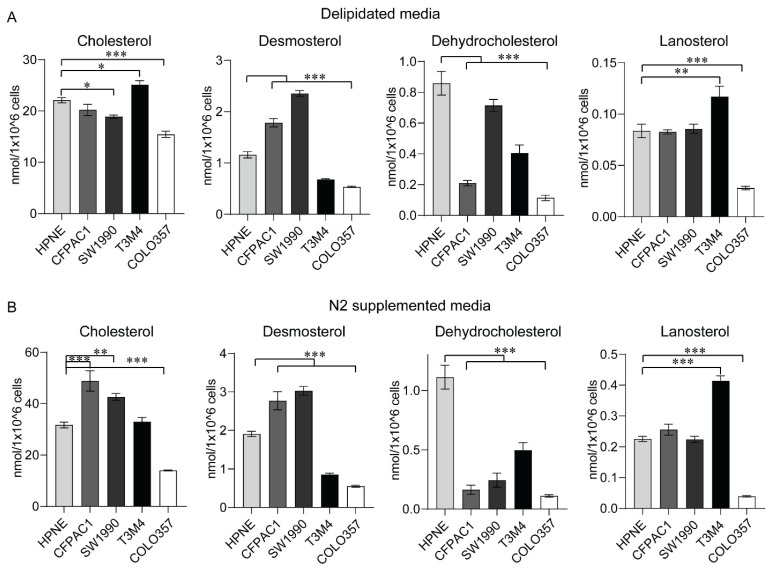
Sterol levels in pancreatic cancer cell lines alter with lipid deprivation. Mean and standard deviation bar plots depicting sterol quantification from immortalized pancreatic epithelial cell line (HPNE) and pancreatic ductal carcinoma cell lines (CFAPC1, SW1990, T3M4, and COLO357) grown in a delipidated medium are shown in panel (**A**), and N2-supplemented medium is shown in panel (**B**). *, **, and *** indicate *p* < 0.01, <0.05, and <0.001, respectively, which were obtained through one-way ANOVA analysis.

As presented in Figure 5, the cellular content of sterol was significantly altered in PC cell lines compared to the HPNE cells, with differences observed in cell lines and media composition. The PC cell line, T3M4, cultured in either delipidated media or N2 media, contained significantly higher cholesterol, desmosterol, and lanosterol than that of the HPNE cells. Though CFPAC1 and SW1990 cells had high desmosterol content when cultured in either media, the cholesterol levels were only elevated in these cells compared to HPNE in N2 media. Another PC cell line, COLO357, utilized in our study showed significantly low sterol contents compared to the HPNE cells. Finally, dehydrocholesterol, which is the precursor metabolite of either cholesterol or desmosterol, was significantly low in all PC cell lines compared to the HPNE cells, indicating the conversion of 7-DHC to cholesterol in PC cells compared to normal HPNE cells.

### 2.6. Patient Survival Is Dependent on Cholesterol Metabolism-Associated Genes in PC

**Figure 6:**
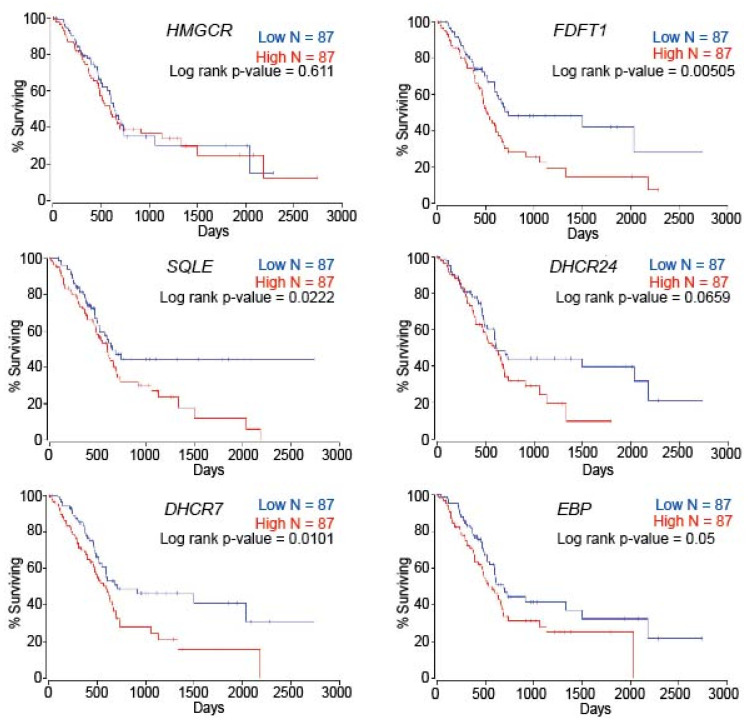
Genes involved in cholesterol synthesis affect patient survival in PDAC. Kaplan–Meier survival curves reflecting the impact of cholesterol metabolic genes expression on patient survival in PDAC.

To evaluate the overall impact of altered cholesterol synthesis on the survival of PC patients, we compared the patient survival relative to the gene expression levels of sterol biosynthesis enzymes. We found that the survival of PC patients declined with an elevated expression of the following cholesterol synthesis pathway genes: *FDFT1*, *SQLE*, *DHCR7*, *DHCR24*, and *EBP* (Figure 6).

## 3. Discussion

Pancreatic ductal adenocarcinoma possesses hyperplastic and neoplastic tumor lesions (Figure 1A) originating spontaneously from the exocrine pancreas [26,27]. Neoplastic, transformed PC relies on a metabolic upsurge, including high cholesterol, to maintain survival and proliferation, which could be compensated through de novo cholesterol synthesis [28]. The dependence of malignant cells on de novo cholesterol biosynthesis is so prominent that the Kandutsch–Russell branch (Figure 1B) of the cholesterol synthesis pathway was first described in cancer cells [29]; thus, metabolically demarcating malignancies from normal tissues. Such demarcation can be revealed through alteration in expression of metabolic genes, as evident from the upregulation of genes involved in cholesterol biosynthesis in PC patient cohorts in our study (Figure 1C,D). Similar comparisons of cholesterol synthesis gene expression patterns between normal and malignant cohorts have been utilized to reveal and target cholesterol synthesis pathway in cancer [30,31]. Our study indicates upregulation of multiple genes that are particularly enzymes involved in cholesterol biosynthesis, i.e., *GGPS1*, *SQLE*, *FDPS*, *HMGCS1*, and *HMGCR,* which were also reported in distinct cancer models [30,31,32,33,34,35,36,37,38]. Such comparisons led to enumeration of the role of individual genes involved in cholesterol synthesis pathway in different cancer models including PC [30,35].

Sterol analysis provides direct evidence of abnormal cholesterol metabolism and often corroborates with sterol gene expression patterns in cancer [39]. In our study, high free cholesterol levels in PC tumors compared to the normal pancreatic tissues reflect two possibilities: accumulation of free cholesterol in tumors through uptake of cholesterol and its precursors from extra tumoral sources, as shown previously [11], or elevated cholesterol biosynthesis within tumors [14]. Based on higher free cholesterol in pancreatic tumors (as shown in Figure 2A), along with the upregulation of cholesterol biosynthesis genes in pancreatic cancer patients, we conclude that the cholesterol biosynthesis pathway (Figure 1C,D) is undoubtedly upregulated in pancreatic cancer patients and in PC tumors when combined with metastatic sites, which could be supported by the notion that cholesterol maintains growth and metastasis in PC [12,13]. However, the lack of significant differences in sterol levels other than free cholesterol in our analysis leaves scope for future studies involving a greater number of samples to address the role of cholesterol metabolic pathway intermediates in PC pathogenesis.

Tumor metabolism is often reflected by abnormal metabolic profiles in nontumoral tissues like plasma [40]. We demonstrate high free cholesterol levels from serum samples in PC (Figure 3A), which could serve as an indication of upregulated cholesterol synthesis in pancreatic tumors. Furthermore, high free cholesterol levels in sera from stage 2b patient samples of pancreatic cancer (Figure 3B) indicate that serum cholesterol levels alter with the progression of pancreatic cancer. Previous studies reported controversial correlations between high serum cholesterol and pancreatic cancer incidence [41,42]. Moreover, recent findings indicate that the impact of cholesterol metabolism on PC would be affected by genomic perturbations prevalent in this cancer [28]. Despite the ambiguity of metabolic perturbations from clinical specimens of PC, murine models have been ideal in recapitulating metabolic deviation in PC. Our sterol analysis from murine PC models also substantiates that sterol metabolites, especially cholesterol and DHC, are high in the pancreas of KPC mice harboring activated *Kras*^G12D^ and *Trp53*^R172H^ compared to the normal pancreas and KC mice (Figure 4), which indicates recapitulation of human pancreatic tumor sterol levels in mice models of PC. Though human pancreatic cancer-derived cell lines recapitulate most tumor cell metabolism in in vitro conditions [43], our analysis using pancreatic tumor cell line models revealed heterogeneity in sterol content of PC cells in comparison to HPNE cells. However, our study revealed higher cholesterol and its precursor levels in most of the PC cell lines in lipid-deprived media, which substantiates high de novo cholesterol synthesis in PC cells.

## 4. Materials and Methods

### 4.1. Chemicals

Unless otherwise noted, all chemicals were purchased from Sigma-Aldrich Co. (St. Louis, MO, USA). HPLC-grade solvents were purchased from Thermo Fisher Scientific Inc. (Waltham, MA, USA). All sterol standards, natural and isotopically labeled, used in this study are available from Kerafast, Inc. (Boston, MA, USA).

### 4.2. Mice and Ethics Statement

We generated and characterized *Kras*^G12D^;*PdxCre* (KC), and *Kras*^G12D^;*Trp*53^R172H^;*PdxCre* (KPC) mouse models. The mice positive for *Kras*, *Trp53,* and *PdxCre* (Floxed *Kras*^G12D^ and *Trp53*^R172H^ (KPC), 20–25 weeks old), as well as mice positive for *Kras* and *PdxCre* (Floxed *Kras*^G12D^ (KC), 40–50 weeks old), and their contemporary littermates (Unfloxed *Kras*^G12D^ (LSLKras^G12D^) of both sexes were utilized for this study [44]. The pancreas of each mouse was resected and flash-frozen in liquid nitrogen. Throughout the study, the animals were humanely treated and subjected to a 12 h of dark/light cycle with food and water ad libitum. Animal studies were performed in accordance with the United States Public Health Service “Guidelines for the Care and Use of Laboratory Animals” under an approved protocol by the University of Nebraska Medical Center’s Institutional Animal Care and Use Committee (IACUC). Pancreatic tissue samples were frozen and kept at −80 °C until sterol extraction. Frozen pancreatic tissue samples were sonicated in ice-cold PBS containing butylated hydroxytoluene (BHT) and triphenylphosphine (PPh3). The aliquots of homogenized tissue were used for sterol and oxysterol extractions and protein measurements. The protein was measured using a BCA assay (Pierce). Sterols were extracted with Folch solution. The organic phase was dried in a speed vacuum, and samples were derivatized with Phenyltriazoledione (PTAD) as described in Section 4.6. Sterol levels were normalized to protein measurements and expressed as nmol/mg protein.

### 4.3. Plasma and Pancreatic Tissue Preparation

Archived and deidentified serum samples were obtained for sterol analysis from the Nebraska Biobank, UNMC, and stored at −80 °C pending analysis. These samples included serum processed from normal (healthy) and pancreatic cancer patient blood samples available through Nebraska Biobank. Serum samples were thawed on ice during the extraction, and 10 mL aliquots were taken for the sterol measurements. Sterols from 10 mL aliquots were extracted without hydrolysis, derivatized with PTAD, and analyzed using LC–MS/MS methods described previously [45]. Frozen pancreatic tumor tissues obtained from PC patients and their adjacent normal pancreatic tissues were procured through Rapid Autopsy Program, UNMC, and processed according to the method described in Section 2.2 for sterol analyses.

### 4.4. Cell Culture and Preparation

Human pancreatic cancer cell lines (CFPAC1, SW1990) and normal pancreatic cell line HPNE were obtained from American Type Culture Collection, whereas T3M4 and COLO357 were obtained from our collaborator. All the cell lines were validated by short tandem repeat profiling (STR) DNA profiling at University of Nebraska Center (UNMC), and before performing experiments, cell lines were tested for mycoplasma contamination. Human pancreatic cancer cell line CFPAC1 was maintained in IMDM, while others, SW1990, T3M4, and COLO357, were maintained in DMEM. In addition, basal medium with low serum and epidermal growth factor was used to maintain the HPNE cell line. All media were supplemented with 10% fetal bovine serum (FBS) and antibiotics (100 U/mL penicillin and 0.1 mg/mL streptomycin) at 37 °C with 5% CO_2_ in a humidified atmosphere.

### 4.5. Metabolite Extraction from Cell Lines

Cells were seeded with an approximate density of 10,000 cells/well into 96-well plates in their regular growth media. After several hours of cellular attachment, the medium was replaced with defined media, which was either delipidated, serum-containing DMEM or N2 supplement medium, and cells were incubated for 48 h. At the end of incubation, Hoechst dye was added, and the plate was imaged on ImageXpress Pico to count the total number of cells per well. After that, the medium was removed, cells were rinsed twice with 1X PBS, 10 mL of antioxidant (butylated hydroxytoluene/sodium tripolyphosphate, BHT/TPP) was added to each well, and the plate was frozen at −80 °C until sterol extraction. For sterol extraction, we added 200 mL of methanol per well and followed the analysis method as described in our previous methodology [46].

### 4.6. LC–MS/MS (SRM) Analyses

Sterols were extracted and derivatized with PTAD as described previously [47] and placed in an Acquity UPLC system equipped with an ANSI-compliant well plate holder coupled to a Thermo Scientific TSQ Quantis mass spectrometer equipped with an APCI source. Then, 5 μL of sample was injected onto the column (Phenomenex Luna Omega C18, 1.6 μm, 100 Å, 2.1 mm × 50 mm) with 100% methanol (0.1% *v*/*v* acetic acid) mobile phase for 1.0 min runtime at a flow rate of 500 μL/min. Natural sterols were analyzed by selective reaction monitoring (SRM) using the following transitions: Chol 369 → 369, 7-DHC 560 → 365, 8-DHC 558 → 363, desmosterol 592 → 560, lanosterol 634 → 602, with retention times of 0.7, 0.4, 0.4, 0.3, and 0.3 min, respectively. SRMs for the internal standards were set to d_7_-Chol 376 → 376, d_7_-7-DHC 567 → 372, d_7_-8-DHC 565 → 370, ^13^C_3_-desmosterol 595 → 563, ^13^C_3_-lanosterol 637 → 605. Final sterol numbers are reported as nmol/mg of protein, and 7-DHC and 8-DHC are reported combined as dehydrocholesterol.

### 4.7. Gene Expression and Survival Analyses

The publicly available, normalized datasets and their corresponding metadata (GSE19650 and GSE32676) were retrieved from Gene Expression Omnibus (GEO) repository [48]. Reactome cholesterol biosynthesis geneset from mSig database was used for the metabolic gene expression profiling across the groups [49,50]. Kaplan–Meier survival analyses corresponding to effects of cholesterol synthesis pathway genes on PDAC patient survival were retrieved using OncoLnc tool (http://www.oncolnc.org, accessed on 19 April 2021).

### 4.8. Statistics

Statistical analyses were performed using GraphPad Prism 9 for Windows and Microsoft Excel. An unpaired, two-tailed *t*-test was performed for individual comparisons between two groups. Welch’s correction was employed when the variances between the two groups were significantly different. Ordinary one-way ANOVA and multiple comparisons with Tukey corrections were performed for comparisons between three or more groups. The *p*-values for statistically significant differences are highlighted in the figure legends.

## 5. Conclusions

The cholesterol biosynthesis pathway plays a significant role in cellular metabolism as it provides cholesterol and other sterol intermediates required for multiple metabolic pathways associated with sterols [41,51]. Our in silico and metabolite analysis revealed the upregulation of cholesterol biosynthesis in PC, which could explain the high cholesterol levels in the plasma of PC patients, which has been of keen interest in cancer as a potential target [52]. For instance, the upregulation of the rate-limiting enzyme HMGCR inevitably enhances cholesterol synthesis in different malignancies, making it a predictable survival marker and target in malignancies [37]. However, targeting HMGCR alone for curtailing the cholesterol synthesis pathway using statins revealed controversial outcomes across malignancies [53]. Thus, identification of cancer type-specific deviations in the cholesterol biosynthesis would be more beneficial, and our study reveals that cholesterol synthesis is a critical target in PC. However, enumeration of ideal targets, models, and their metabolic outcomes is warranted before optimal preclinical and clinical targeting of cholesterol synthesis pathway could be achieved in PC. In the current study, we applied multiple modalities to identify the upregulation of the cholesterol synthesis pathway in PC and confirmed that clinical, in vivo, and in vitro models of PC could serve as complementary models to decipher the exact functional involvement of cholesterol biosynthesis in PC. Furthermore, our study also demonstrates the poor prognosis associated with the cholesterol metabolic genes in PC. However, future studies using large patient cohort samples are warranted to discriminate the prospective role of individual cholesterol biosynthesis steps in PC. Overall, our study reflects the potential for individual PC models and applicability of in silico and sterol analysis in identifying potential targets in the cholesterol synthesis pathway in PDAC.

## Data Availability

The data presented in this study are available on request from the corresponding author. The data are not publicly available due to ethical.
